# Predictors of Cognitive Decline in Older Adult Type 2 Diabetes from the Veterans Affairs Diabetes Trial

**DOI:** 10.3389/fendo.2016.00123

**Published:** 2016-09-08

**Authors:** Mark B. Zimering, Jeffrey Knight, Ling Ge, Gideon Bahn, the VADT Investigators, Jeremy Soule

**Affiliations:** ^1^Medical Service, Department of Veterans Affairs New Jersey Health Care System, Lyons, NJ, USA; ^2^Rutgers-Robert Wood Johnson Medical School, New Brunswick, NJ, USA; ^3^National Center for PTSD, VA Boston Healthcare System, Boston MA, USA; ^4^Boston University School of Medicine, Boston, MA, USA; ^5^Hines Veterans Affairs Hospital, Hines, IL, USA

**Keywords:** cognitive decline, type 2 diabetes mellitus, risk factors, blood pressure, diabetes duration

## Abstract

**Aims:**

Cognitive decline disproportionately affects older adult type 2 diabetes. We tested whether randomized intensive (INT) glucose-lowering reduces the rate(s) of cognitive decline in adults with advanced type 2 diabetes (mean: age, 60 years; diabetes duration, 11 years) from the Veterans Affairs Diabetes Trial.

**Methods:**

A battery of neuropsychological tests [digit span, digit symbol substitution (DSym), and Trails-making Test-Part B (TMT-B)] was administered at baseline in ~1700 participants and repeated at year 5. Thirty-seven risk factors were evaluated as predictors of cognitive decline in multivariable regression analyses.

**Results:**

The mean age-adjusted DSym or TMT-B declined significantly in all study participants (*P* < 0.001). Randomized INT glucose-lowering did not significantly alter the rate of cognitive decline. The final model of risk factors associated with 5-year decline in age-adjusted TMT-B included as significant predictors: longer baseline diabetes duration (beta = −0.028; *P* = 0.0057), lower baseline diastolic blood pressure (BP; beta = 0.028; *P* = 0.002), and baseline calcium channel blocker medication use (beta = −0.639; *P* < 0.001). Higher baseline pulse pressure was significantly associated with decline in age-adjusted TMT-B suggesting a role for both higher systolic and lower diastolic BPs. Baseline thiazide diuretic use (beta = −0.549; *P* = 0.015) was an additional significant predictor of 5-year decline in age-adjusted digit symbol score. Post-baseline systolic BP-lowering was significantly associated (*P* < 0.001) with decline in TMT-B performance. There was a significant inverse association between post-baseline plasma triglyceride-lowering (*P* = 0.045) and decline in digit symbol substitution task performance.

**Conclusion:**

A 5-year period of randomized INT glucose-lowering did not significantly reduce the rate of cognitive decline in older-aged adults with type 2 diabetes. Systolic and diastolic BPs as well as plasma triglycerides appeared as modifiable risk factors of the rate of cognitive decline in older adult type 2 diabetes.

## Introduction

Diabetes is associated with a twofold increased risk for age-related cognitive decline or dementia (1, 2). Increasing life expectancy and the current global epidemic of type 2 diabetes mellitus (T2DM) (3) may cause a sharp increase in the numbers of older T2DM adults suffering with cognitive impairment or dementia (4) during the next several decades. Yet the key risk factors and underlying mechanisms contributing to accelerated cognitive aging in adult T2DM remain largely undefined. Type 2 diabetes and age-related cognitive decline share several underlying risk factors, including obesity, hypertension, and hyperlipidemia. Yet, few clinical trials have addressed whether randomization to intensive (INT) glycemic control may slow the rate of cognitive decline (5). The Veterans Affairs Diabetes Trial (VADT) randomized 1791 older adults with T2DM to standard (STD) or INT glycemic treatment (6). Similar desirable mean blood pressure (BP) and lipid levels were achieved in the majority of STD- or INT-treated participants (6). After an average of 6 years of study treatment, the primary VADT cardiovascular disease (CVD) outcome did not differ significantly according to original randomized glycemic treatment assignment (6). The current study is from a planned secondary analysis to the VADT designed to test whether INT glycemic treatment slows cognitive decline in older adult T2DM. All study participants underwent a battery of neuropsychological tests to assess cognitive function in several overlapping domains, e.g., working memory, processing speed, and executive function. The neuropsychological tests, digit span (DS), digit symbol substitution task (DSym), and Trails-making Test-Part B (TMT-B) were administered at baseline and again at the year 5 study visit in all available participants.

## Subjects and Methods

### Study Participants

The study design and clinical inclusion and exclusion criteria for the main VADT have been previously reported (6). Written informed consent to the local IRB-approved protocol was provided by all participants at each of the 20 participating study sites. Eligible patients without significant renal insufficiency or congestive heart failure were randomly assigned to STD vs. INT glycemic treatment. All participants were older than 40 years, and 96% were men. Among 1791 randomized participants, 1678 completed all three neuropsychological tests at baseline. One hundred thirty-six participants had terminated study participation prior to year 5, and did not undergo repeat neuropsychological testing. An additional three hundred eighty-three participants did not undergo repeat (year 5) neuropsychological testing. Participants who were lost to year 5 neuropsychological testing follow-up did not differ significantly in their baseline age, systolic or diastolic BP, or randomized glycemic study treatment assignment compared to those who completed year 5 testing. Participants who were lost to neuropsychological follow-up testing had slightly longer baseline duration of diabetes (12.0 vs. 11.3 years) and a higher percentage had experienced a prior CVD event at baseline study entry (44 vs. 38%; *P* = 0.014) compared to participants who completed both baseline and year 5 neuropsychological testing.

### Medications

As previously reported, all patients were receiving antidiabetic medications at baseline study entry, including oral agents and/or insulin (6). Randomized glycemic treatment in the STD or INT arms involved the use of similar classes of antidiabetic medications, but at different doses. The thiazolidinedione (TZD) rosiglitazone was prescribed for most patients randomized to INT treatment. Baseline anti-hypertensive medication classes included angiotensin-converting enzyme (ACE) inhibitors, angiotensin receptor blockers (ARBs), calcium channel blockers (CCB), and thiazide diuretics.

### Blood Pressure and Laboratory Measures

Blood pressure was recorded in study clinics while patients were seated and after allowing a 5-min period of rest. The median value of the three consecutive determinations, computed separately for systolic and diastolic BPs, was used for analysis. Laboratory measures, including urinary microalbumin, plasma hemoglobin A_1_c, urine creatinine, plasma total cholesterol, triglycerides, and high-density lipoprotein (HDL) cholesterol, were determined by standardized methods, as previously described (6).

### Neuropsychological Test Measures

Three standardized neuropsychological tests were administered at baseline and repeated at the year 5 study visit for all able, available VADT study participants. The tests were DS and digit symbol (DSym) subtests, both from The Wechsler Adult Intelligence Scale-III (7), and the TMT-B (8, 9). These three tests collectively are neurocognitive measures of auditory and visual attention, concentration, mental control, short-term auditory memory, verbal working memory, verbal memory span, cognitive processing speed, cognitive flexibility, task switching, sequencing, spatial organization, visual pursuit, and executive cognitive function. For the DS test, the subject listens to a string of verbally presented numbers, then repeats them back. The difficulty increases as the digit string lengthens. The digits have no logical relationship to each other. DS Forward requires a repetition of the number sequence exactly as presented; DS Backwards requires the number sequence to be repeated in reverse order (e.g., response for “1-8-3-7” is “7-3-8-1”). The test is discontinued when two repetition attempts are failed at a particular string length (e.g., six digits cannot be repeated correctly). The longest string length successfully repeated is then recorded as the final score. DS Forward and DS Backwards scores are summed to a final DS raw score. DS measures short-term auditory memory, attention, concentration, and working memory. Verbal working memory is necessary for everyday tasks involving recall of sequences of information (e.g., telephone numbers, understanding long and complex sentences).

DSym is a matching-to-sample task where key symbols are uniquely associated with a number from 1 to 9. An 8.5 × 11″ response page presents a row of boxes showing the symbol–number combinations to be used as the reference for the task. Below the reference key row is a practice sample, followed by rows of boxes containing a number, with a blank area to write in the associated symbol. The test is timed; the goal is to complete as many symbol–number combinations as possible before time expires. Higher scores are associated with better working memory, strategy/planning, psychomotor competence, visual scanning, and efficiency of new learning. DSym is a multi-factorial test that is sensitive to decreased cognitive functioning secondary to changes in brain functioning.

Trails-making Test-Part B involves drawing, with a pencil on paper, a trail between 25, encircled numbers and letters that are randomly distributed across the 8.5 × 11″ response page (“connect-the-dots”). TMT-B requires that the subject alternate between numbers and letters (i.e., draw a line from 1 to A, A to 2, 2 to B, etc.) as quickly as possible without making sequencing errors. The test is timed and the final score represents the amount of time required to draw a line connecting all 25 circles. TMT-B is also a multi-factorial test that is sensitive to decreased cognitive functioning secondary to changes in brain functioning. For each neuropsychological test, the individual raw score was transformed to an age-specific, scaled score using published normative tables (7, 10).

Change in scaled neurocognitive test scores was calculated as the difference between year 5 and baseline scaled scores. A negative value for the difference is indicative of a decline in cognitive test performance. Since higher raw score in the TMT-B (seconds required for completion) is indicative of worse cognitive performance, transformation of the TMT-B raw score to a scaled score involves the use of an inverse scaling factor. Post-baseline risk factor change (e.g., systolic BP) was calculated as the difference between the year 5 and baseline mean level. Raw cognitive test scores that appeared as significant outliers, e.g., DS > 30 or DSym > 133, were considered as unreliable and treated as missing data. In several patients who were either unable to hold a pen/pencil, or had severe visual impairment, i.e., were legally-blind, the TMT-B raw score (>300 s) was treated as missing data. The neuropsychological test data were continually monitored by VA Cooperative Study Coordinating Center staff during the study trial, and lay staff at all study sites were provided with additional training in the administration of neuropsychological test battery at regularly scheduled study meetings.

### Statistics

Multivariable regression analysis was used to model the association between baseline risk factors and the 5-year change in scaled DS, digit symbol, or TMT-B. Thirty-seven potential risk factors grouped according to their relatedness, e.g., demographic (race, ethnicity, age, gender, education level), anthropometric (weight, BMI, waist–hip ratio, height), atherosclerosis (smoking, systolic or diastolic BP, total cholesterol, LDL cholesterol, HDL cholesterol, triglycerides), thrombosis (baseline aspirin use, PAI-1, fibrinogen), medical co-morbidities (hypertension, prior CVD event, nephropathy, serum creatinine, microalbuminuria), glucose-lowering medications (insulin, sulfonylureas, TZD, metformin), BP-lowering medications (CCB, thiazide diuretic, ACE inhibitor or ARB), lipid-lowering medications (statins, fibrates, fish oil) or diabetes-specific variables (diabetes duration, any hypoglycemia, any post-baseline, or severe post-baseline hypoglycemia) were evaluated (in multivariable regression analysis) for association with 5-year change in scaled cognitive test score(s). Risk variable(s) significantly or nearly significantly (*P* < 0.10) associated with 5-year change in age-adjusted, scaled cognitive test score(s) were included (together with randomized glycemic treatment group) as covariates in final regression modeling of risk factors associated with change in cognitive test score. Backward elimination was used to obtain the best-fit model using an α level of less than 0.05 as the cutoff for variable retention in the final model.

## Results

### Baseline Scaled Cognitive Test Score(s) by Randomized Treatment Assignment

Baseline clinical characteristics previously reported in the study population did not differ significantly by randomized glycemic treatment group, including mean (SD): age 60.4 (8.7) years, body mass index 31.3 (3.5) kg/m^2^, hemoglobin A_1_c 9.4 (2.0)%, diabetes duration 11.4 (7.5) years, systolic BP 132 (17) mmHg, diastolic BP 76 (10) mmHg, or total cholesterol concentration 184 (47) mg/dL (6). Forty percent of patients reported a prior cardiovascular event, 72% had baseline hypertension, and 17% of participants were current smokers at baseline study entry (6). There was no significant difference in baseline educational attainment in study participants randomized to STD vs. INT glycemic treatment (Table 1). There was no significant difference in the baseline scaled DS, digit symbol substitution, or TMT-B score in patients assigned to STD or INT glycemic control (Table 1).

**Table 1:** **Baseline clinical characteristics in study subjects by randomized treatment**.

	STD	INT	*P*-value
Education			
Less than high school	29	36	0.39
Some high school	63	68	0.66
GED or high school graduate	255	265	0.66
Vocational or technical school	43	47	0.67
Some college	322	293	0.24
College graduate	116	105	0.46
Some graduate school	18	22	0.53
Graduate/professional degree	50	56	0.56
Total	896	892	
Scaled digit span	10.18 ± 3.04 (*n* = 887)	10.16 ± 2.99 (*n* = 877)	0.92
Scaled digit symbol	8.84 ± 3.34 (*n* = 886)	8.75 ± 3.28 (*n* = 873)	0.58
Scaled Trails B	8.60 ± 2.31 (*n* = 844)	8.74 ± 2.30 (*n* = 834)	0.21

*STD (standard), INT (intensive) glycemic treatment*.

*Results are *N* (for Education level) or mean ± SD (for scaled cognitive test scores)*.

### Five-Year Change in Scaled Cognitive Test Scores

**Figure 1:**
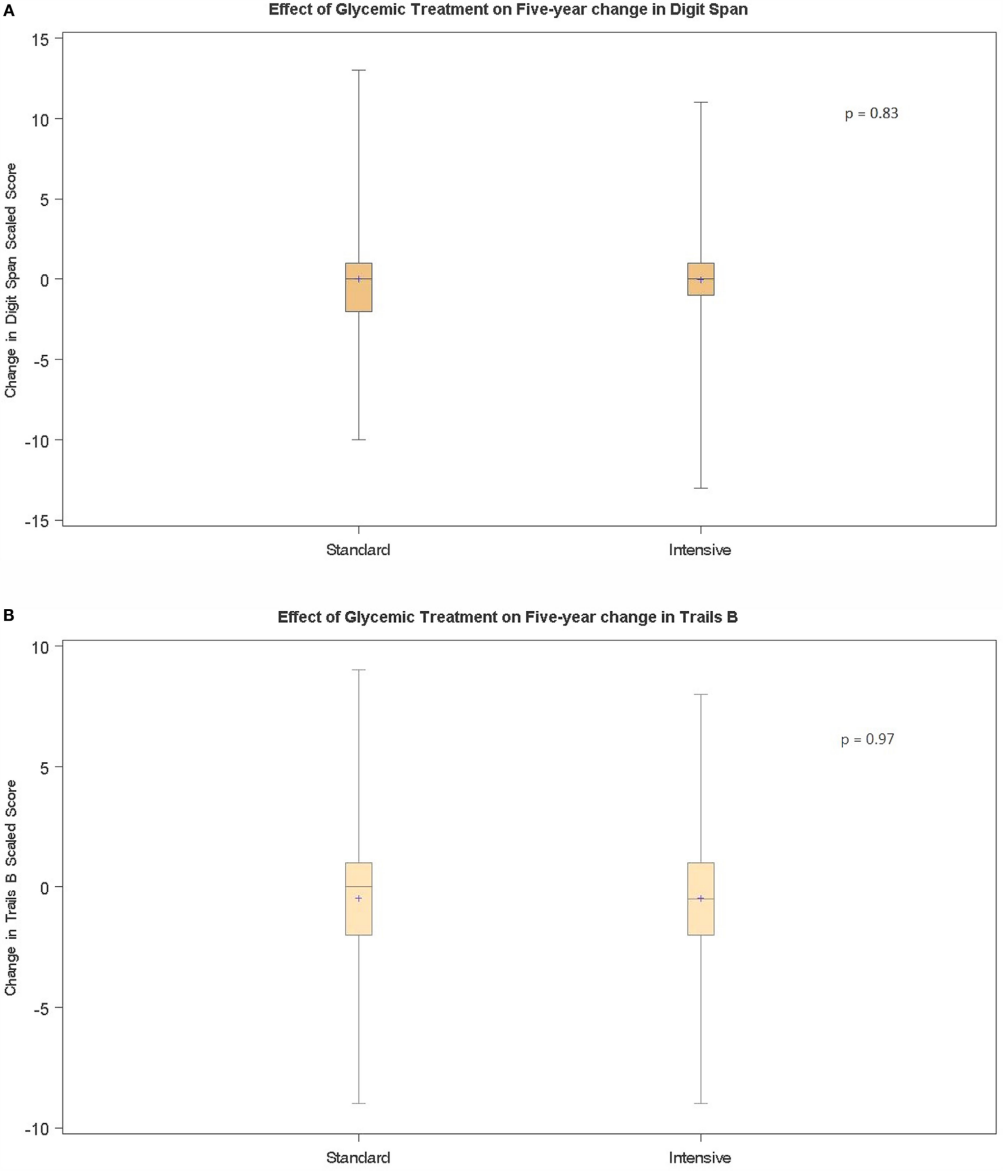

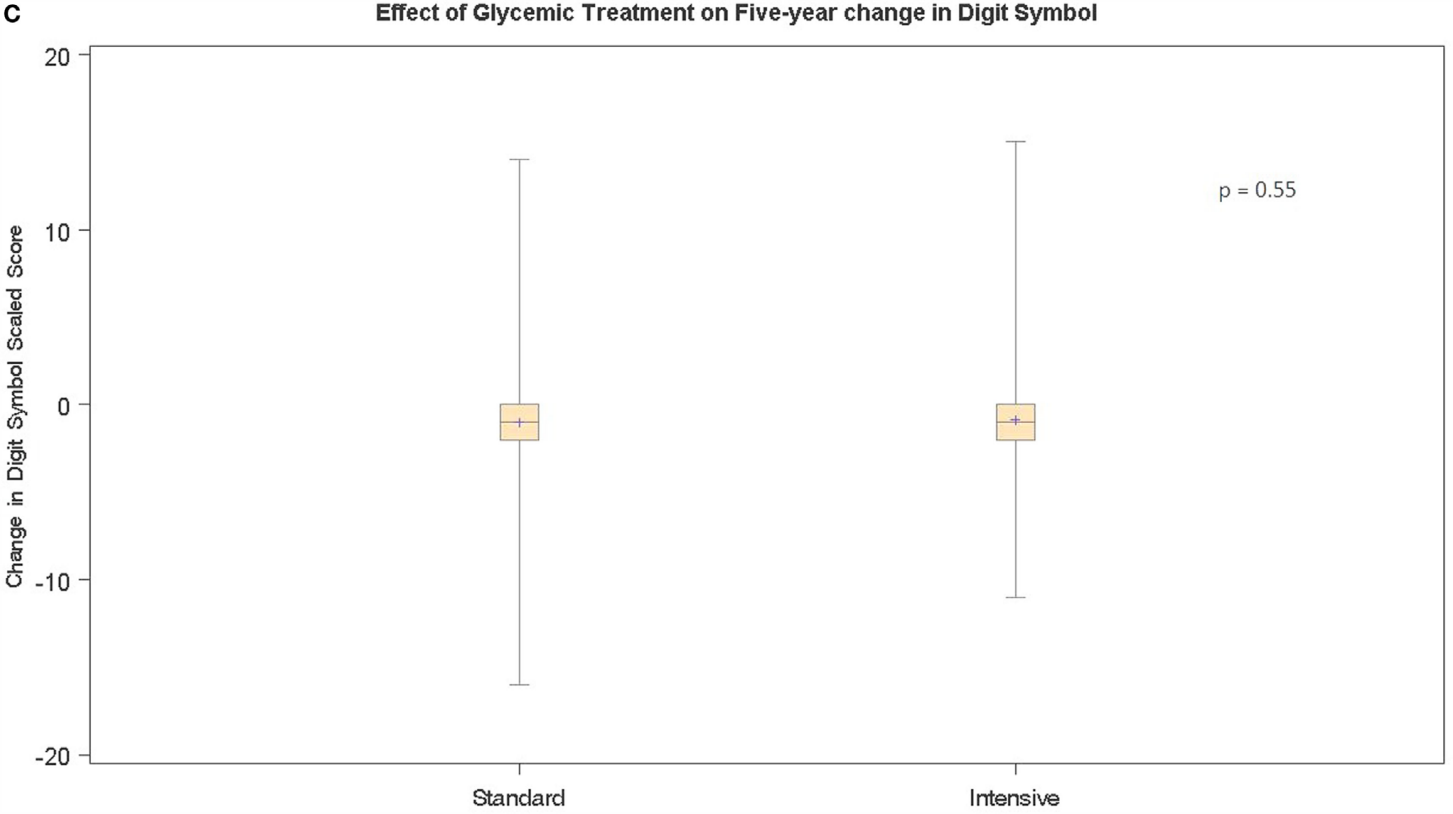
**Box–whisker plot of the distribution of 5-year change in (A) digit span, (B) Trails making Test-Part B, or (C) Digit Symbol age-adjusted test score in standard vs. intensive glucose-lowering treatment groups**. **(A–C)** *P*-value(s) from *T*-test comparing mean scaled score in standard vs. intensive glucose-lowering treatment group. Boxes represent first and third quartiles, horizontal line denotes median value, whiskers represent minimum and maximum values. **(A)** *N* = 1174; **(B)** *N* = 1155; **(C)** *N* = 1114.

We next computed the mean (and 95% confidence intervals) for the scaled DS, digit symbol, and Trails B test scores obtained at baseline and repeated at the 5-year study visit by a subset of all participants (Table 2). There was no significant difference (−0.003) in the mean scaled DS score (between the baseline and the 5-year interval measurement) in participants who completed the DS at both study intervals (Table 2). The mean scaled digit symbol score declined significantly (−0.92; *P* < 0.001, Table 2) and the mean scaled TMT-B declined significantly (−0.488; *P* < 0.001, Table 2) between the baseline and 5-year interval measurements. These results are consistent with Yeung et al. (11) who reported that executive function (Trails B) and processing speed (digit symbol) were affected by type 2 diabetes to a significantly greater extent than was recall. The distribution of change in scaled cognitive test score in all study participants performance by randomized treatment assignment group is illustrated in Figures 1A–C. There were no significant differences in the mean change (over the 5-year period) in scaled DS (Figure 1A, *P* = 0.83), scaled digit symbol (Figure 1B, *P* = 0.97) or scaled TMT-B (Figure 1C, *P* = 0.55) test score between participants randomized to intensive (INT) vs. STD glycemic treatment.

**Table 2:** **Mean change in 5-year scaled cognitive test score**.

Test	Baseline	Year 5	Mean change^a^	95% CI	*N*	*P*-value^^^
Digit span	10.279	10.277	−0.003	−0.153 to 0.148	1174	NS
Digit symbol	8.995	8.080	−0.92	−1.086 to −0.745	1155	<0.001
Trails B	8.703	8.226	−0.48	−0.622 to −0.331	1114	<0.001

*^a^Change is (Year 5 – baseline) scaled score, NS, not significant*.

*^^^*P*-value from *T*-test, compared to no change*.

*N = 1174 (Digit span), N = 1155 (Digit symbol), N = 1114 (Trails B)*.

### Association between Groups of Related Risk Factors and 5-Year Cognitive Decline

There was no significant association between 5-year change in any of the three cognitive tests and the following baseline risk variables: body mass index, waist–hip ratio, gender, smoking, education, serum creatinine, microalbuminuria, plasma total cholesterol, LDL cholesterol, HDL cholesterol or triglyceride concentration, any baseline hypoglycemia, (any or severe) post-baseline hypoglycemia, baseline use of insulin, TZD, ACE inhibitor, ARBs, or fish oil medication. In nested, multivariable regression analyses that tested groups of related variables, higher baseline systolic BP, higher baseline pulse pressure, higher plasma fibrinogen, baseline aspirin use, baseline metformin use, baseline history of prior CVD event was each significantly associated with 5-year decline in TMT-B score (Table S1 in Supplementary Material). Baseline aspirin or sulfonylurea use was significantly or inversely significantly associated, respectively, with the rate of decline in scaled DSym test score (Table S2 in Supplementary Material). There was a non-significant trend (*P* = 0.068) of an association between baseline statin use and 5-year decline in Trails making B test score (Table S1 in Supplementary Material), a non-significant trend (*P* = 0.071) of an association between baseline statin use and 5-year decline in DS test score (not shown in Supplementary Material) and a non-significant trend (*P* = 0.075) of an association between baseline fibrate use and 5-year improvement in DSym test soccer (Table S2 in Supplementary Material).

### Final Model of Risk Factors Associated with Decline in Cognitive Test Performance

The final model of risk factors associated with 5-year decline in scaled DS test performance included as significant predictors: African-American race (*P* < 0.001), and baseline thiazide diuretic use (*P* = 0.036) (Table 3). The final model of risk factors associated with 5-year decline in scaled TMT-B included as significant predictors: diabetes duration (*P* = 0.006), diastolic BP (*P* = 0.002), and baseline CCB medication use (*P* < 0.001) (Table 3). The final model of risk factors associated with 5-year decline in scaled digit symbol substitution task included as significant predictors: diabetes duration (*P* = 0.034), baseline CCB medication use (*P* = 0.011), and baseline thiazide diuretic use (*P* = 0.015) (Table 3).

**Table 3:** **Final model of risk factors associated with 5-year decline in (A) digit span, (B) Trails-making Part B, and (C) digit symbol test performance**.

Parameter	Estimate	SE	*P*-value
**(A) Digit span**			
Treatment (INT vs. STD)	−0.026	0.153	0.863
African-American (yes/no)	−0.807	0.209	<0.001
Thiazide diuretic (yes/no)	−0.425	0.202	0.036
**(B) Trails B**			
Treatment (INT vs. STD)	0.0024	0.147	0.987
Diabetes duration (years)	−0.028	0.010	0.006
Diastolic BP (mmHg)	0.028	0.0091	0.002
CCB (yes/no)	−0.639	0.184	<0.001
**(C) Digit symbol**			
Treatment (INT vs. STD)	0.067	0.172	0.697
Diabetes duration (years)	−0.025	0.012	0.034
CCB (yes/no)	−0.557	0.217	0.011
Thiazide diuretic (yes/no)	−0.549	0.225	0.015

*(A) *N* = 1159*.

*(B) *N* = 1099; BP, blood pressure; CCB-calcium channel blocker*.

*(C) *N* = 1139*.

### Association between Post-Baseline Risk Factors and Decline in Cognitive Test Performance

Post-baseline systolic BP-lowering was significantly associated (*P* < 0.001) with decline in TMT-B performance (Table 4); and post-baseline diastolic BP-lowering was significantly inversely associated (*P* = 0.003) with decline in TMT-B performance (Table 4). Post-baseline pulse pressure-lowering was significantly associated (*P* < 0.001) with decline in TMT-B performance (Table 4). There was a significant inverse association between post-baseline plasma triglyceride-lowering (*P* = 0.045) and decline in digit symbol substitution task performance (Table 4). There were no significant associations between post-baseline plasma total cholesterol-lowering and 5-year decline in DS (*P* = 0.536), DSym (*P* = 0.706), or TMT-B (*P* = 0.244) test performance.

**Table 4:** **Association between post-baseline modifiable risk factor level and change in scaled cognitive test performance**.

Parameter	Estimate	SE	*P*-value
**(A) Trails B**			
Change in syst BP (mmHg)	0.018	0.0050	<0.001
Change in dias BP (mmHg)	−0.026	0.0086	0.003
**(B) Trails B**			
Change in pulse press (mmHg)	0.018	0.0051	<0.001
**(C) Digit symbol**			
Change in plasma			
Triglyceride (mg/dL)	−0.0013	0.00064	0.045

*(A) *N* = 1106*.

*(B) *N* = 1109*.

*(C) *N* = 1147; syst(olic), dias(tolic) blood press(ure)*.

## Discussion

The current findings are the first to suggest that a 5-year period of INT glucose-lowering did not significantly slow the rate of cognitive decline experienced by older adult male veterans with T2DM. These findings are in agreement with results from the ACCORD-MIND study in which three and one-third years of randomized INT glucose-lowering did not substantially alter the trajectory of cognitive decline in older adult men and women with advanced type 2 diabetes (5). Since 5–6 years of randomized INT glucose-lowering (in the VADT) did not significantly reduce cardiovascular event occurrence (6) or alter the rate of cognitive decline, these data do not support INT glucose-lowering as a primary therapeutic approach to slowing cognitive decline in older adult men with advanced T2DM.

Prior studies have suggested a long-lasting legacy effect of midlife systolic BP elevation on the rate of cognitive decline among older, non-diabetic adults (12, 13). The present finding of significant associations between baseline systolic BP, baseline pulse pressure or the baseline use of specific anti-hypertensive medication classes, and accelerated rate(s) of decline in working memory, processing speed, and executive function are consistent with a long-lasting “legacy” effect from elevated systolic BP or elevated pulse pressure on the promotion of accelerated cognitive decline in adult T2DM. CCB and thiazide diuretics are known to substantially lower systolic BP (14) and were likely used (in the VADT) principally as add-on medications in patients having difficult-to-control or refractory baseline hypertension. In support of this possibility, African-American patients who are prone to higher rate(s) of systolic hypertension were significantly more likely to have experienced a decline in working memory (scaled DS test score) compared to non-Hispanic white or Hispanic patients.

Systolic hypertension is characterized by elevated systolic and decreased diastolic BP leading to a widened pulse pressure, i.e., the difference between systolic and diastolic BPs. Systolic hypertension frequently accompanies normal aging (15) and is thought to be due at least (in part) to age-related, loss of elastic recoil in large arteries (15). The biologic mechanisms underlying an association between systolic hypertension and accelerated cognitive decline in older adult advanced type 2 diabetes are largely unexplored and beyond the scope of the present study. Yet results from previous longitudinal studies conducted in non-diabetic populations (15–17) suggest that elevated systolic BP (even in young adults) correlated with white matter and gray matter brain structural changes that are early markers of decreased cognitive performance (18, 19). One possible mechanism by which increased arterial stiffness may contribute to cognitive decline is via excessive mechanical force applied to vascular endothelial cells. Studies in animal models or *in vitro* cell systems suggest that endothelial dysfunction resulting from excessive mechanical force causes altered expression of proteases (20), increased vascular permeability (21), or increased endothelial adherence and transmigration of inflammatory cells (22).

Our data suggest an important vascular component to the risk for cognitive impairment in older adult diabetes, 72% of whom had baseline hypertension. For example, in our preliminary multivariable regression analyses, several traditional risk factors associated with stroke and CVD (e.g., baseline aspirin use, plasma fibrinogen) were associated with accelerated rates of decline in processing speed and executive function. Our finding that baseline duration of diabetes was a strong, significant predictor of accelerated decline(s) in processing speed and executive function is consistent with results from other studies (23, 24). We were unable to demonstrate a significant association between any or serious, post-baseline hypoglycemia and accelerated rate of cognitive decline among VADT participants. Even though the total number of hypoglycemia episodes was substantially increased in the INT treatment group, relatively few INT- vs. STD-treated patients (8.5 vs. 3.1%) experienced documented serious hypoglycemia (6).

The current data suggest a complex relationship between post-baseline BP-lowering and the rate of decline in executive function. According to our regression model, each 10 mmHg reduction in post-baseline systolic BP (or pulse pressure) was associated with an 18% decline in scaled TMT-B score. In a VADT patient who was 60 years or older at baseline study entry, an 18% decline in age-adjusted TMT-B score would correspond to advancing cognitive age by ~4 years. Our model predicts that post-baseline diastolic BP-lowering was simultaneously protective against decline in scaled TMT-B score and that maintaining an adequate pulse pressure (during randomized treatment) could mitigate against accelerated cognitive decline. Of interest, the mean pulse pressure in VADT patients assigned to STD or INT glycemic treatment was roughly 56 mmHg both at baseline and at the year 6 follow-up visit (6). Several large randomized clinical trials in non-diabetic populations suggest that, when aggressive systolic BP-lowering was undertaken in older-age hypertensive adults, it did not translate into a consistent reduction in the rate of occurrence of either dementia or substantial cognitive impairment (25). In the ACCORD study of older adult T2DM, INT BP-lowering that targeted systolic BP to a level less than 120 mmHg was associated with significant reduction(s) in total brain volume without an observable effect on the rate of cognitive decline (26). Taken together with the present findings, these results suggest that overly aggressive therapeutic systolic BP-lowering should not be undertaken without compensatory reduction in diastolic BP. Cerebral autoregulation normally ensures constant cerebral blood flow in the setting of declining systolic BP via changes in local brain arteriolar resistance. It is possible, however, that excessive therapeutic narrowing of the pulse pressure may lead to non-pulsatile blood flow that is known to predispose to accelerated atherosclerosis (27) and microvascular endothelial dysfunction (28).

Our novel finding that post-baseline plasma triglyceride-lowering was associated with a significant protection against decline in cognitive processing speed suggests an additional important modifiable risk factor for slowing accelerated cognitive aging in T2DM. In our regression model, each 50 mg/dL reduction in plasma triglycerides was associated with a corresponding 6.5% improvement in digit symbol substitution scaled score. Based on age-specific DSym normative values, a 6.5% improvement in DSS scaled score is predicted to have had an effect equivalent to delaying cognitive aging by nearly 4 years among higher baseline-functioning patients who were 65 years or older at baseline VADT randomization. Of interest, VADT patients randomized to INT or STD treatment experienced mean reductions in plasma triglyceride level of 50 mg/dL (INT) or 64 mg/dL, respectively (6) consistent with an overall cognitive benefit from randomized VADT treatment. By contrast, in the ACCORD study of INT lipid-lowering, three and one-third years of randomized fibrate treatment alone did not significantly alter the rates of decline in processing speed (DSS) or in total brain volume (26). More study is needed to determine whether a longer treatment period (60 vs. 40 months), higher baseline mean plasma triglyceride level (213 vs. 162 mg/dL), or larger post-baseline reduction in mean plasma triglyceride level (57 vs. 30 mg/dL) in VADT participants vs. ACCORD lipid-lowering study participants may have contributed to differences in cognitive study outcomes between the two clinical trials. Epidemiologic data support an association between higher triglyceride level and the incidence of ischemic stroke (29) that may also contribute to cognitive decline in high-risk older diabetic populations. Although INT glucose-lowering *per se* did not alter the rate of cognitive decline in the VADT, substantial and sustained glucose-lowering experienced by both INT- and STD-treated VADT patients may have translated to beneficial slowing of the rate of cognitive decline (in part) via its known favorable effects on very-low-density-lipoprotein metabolism (30).

Our study findings are applicable to older men (mean age 60.4 years) with advanced T2DM (mean duration 11.4 years), highly prevalent hypertension and underlying CVD, i.e., 40% of participants reported a prior CVD event at baseline study entry (6). Strengths of our study included the long treatment interval between baseline and follow-up cognitive performance tests that not only maximized the chance of detecting a true biological change in cognition among individual study participants, but also minimized the possibility of bias due to a “practice effect” on test performance results, as reported for the TMT-B (10). A limitation of our study is the lack of generalizability of the findings to women who are known to experience a higher rate of age-associated dementia or severe cognitive decline (4). The effect(s) of post-baseline BP- or triglyceride-lowering on rate(s) of change in cognitive function must be interpreted with caution since they were derived from *post hoc* exploratory analyses. More randomized study is needed to validate these results and determine optimal treatment targets for systolic BP, diastolic BP, pulse pressure, or plasma triglycerides associated with delaying the rate of accelerated cognitive aging in older adult T2DM.

Statins are among the most widely prescribed medications having proven benefit in reducing cardiovascular event occurrence in middle-aged and older diabetic and non-diabetic populations (31). Recent concerns about statin intolerance (31) have been focused (in part) on reports of statin-induced cognitive dysfunction (32). Yet few data exist in vulnerable older adult type 2 diabetic populations regarding a possible relationship between statin use and accelerated cognitive decline. The present study was not specifically designed to evaluate the effect of statins on cognitive function. Although the borderline significant associations between baseline statin use and accelerated 5-year decline(s) in executive function and recall are of interest, they do not necessarily imply causality. Rather they likely reflect confounding by indication, i.e., higher baseline prevalence(s) of coronary and cerebrovascular atherosclerotic disease among baseline statin-users. Several recent reports suggest that large-vessel cerebrovascular atherosclerosis, e.g., carotid stenosis, alters cerebral hemodynamics resulting in declines in cognitive function (33, 34). Carotid stenosis interacted significantly with impaired cerebral microvascular reactivity (which increases in diabetes) (34) leading to significantly greater declines in executive function, memory, and attention (33, 34).

In summary, our novel findings add to accumulating evidence suggesting that INT glucose-lowering alone should not be undertaken solely for the purpose of reducing the rate of cognitive decline in older-aged adults with type 2 diabetes. Our preliminary results are the first to suggest that systolic BP-lowering, when it is undertaken in older-age individuals (compared to in midlife), has complex effects on the rate of cognitive decline, but that taken together with attention to plasma triglyceride-lowering may provide fruitful avenue(s) for future research aimed at slowing the rate of decline in processing speed (and preserving independence) in older adult T2DM.

## Author Contributions

MZ analyzed data and wrote the paper; JK analyzed data; LG analyzed data; GB analyzed data; and VADT Investigators helped design the study, and performed or supervised data collection. (VADT authors are listed in Presentation 1 in Supplementary Material).

## Conflict of Interest Statement

The authors declare that the research was conducted in the absence of any commercial or financial relationships that could be construed as a potential conflict of interest.
