# Supplementary material for: Predictors of Cognitive Decline in Older Adult Type 2 Diabetes from the Veterans Affairs Diabetes Trial
Source: Front Endocrinol (Lausanne). 2016 Sep 8;7:123. doi: 10.3389/fendo.2016.00123 (PMC5015004; doi:10.3389/fendo.2016.00123)
Supplement: Supplementary file 1 [file presentation_1.pdf]

<u>VADT STUDY SITE INVESTIGATORS & NURSES</u>: Charleston : Jeremy Soule, M.D., Susan Caulder, R.N., M.S.N, C.D.E., Clare Pittman, R.N., M.S., C.D.E., Omayra Alston, Ronald K. Mayfield, M.D.*, Greg Moffitt, M.D.*, Julius Sagel, M.D.*, Frank Sanacor, Pharm.D.* Elizabeth Ganaway, R.N.*; Miami: Jennifer Marks, M.D., Lorraine Okur, R.N., Lucille Jones, R.N., Hermes Florez, M.D., Donna Pfeifer, PhD, ARNP, APRN, BC, Luis Samos, M.D,Andrew L. Taylor, M.D.*; Lyons/East Orange: Mark B. Zimering, M.D., Adilia Sama, R.N.,Frances Rosenberg, R.N.,Heidi Garcia, R.N., Norman Ertel, M.D, Leonard Pogach, M.D., John J. Shin, M.D., Felice Caldarella, M.D.*, Constantino Carseli, M.D.*Mamta Shah, M.D.* ; Fresno: Paulette Ginier, M.D., F.A.C.P., George Arakel, M.D., Yangheng Fu, M.D. *, Don Tayloe, M.D., Jack E. Allen, R.N.*, Elizabeth Fox, M.S.N, C.N.S, NP-C, C.D.E*, Paula G. Hensley, R.N.*.; Hines: Nicholas Emanuele, M.D., Kathleen Kahsen, R.N., C.D.E., Patricia Linnerud, R.N., M.S., Lily Agrawal, M.D., Nasrin Azad, M.D.; Houston : Marco Marcelli, M.D., Glenn R. Cunningham, M.D., Natalie M. Nichols, L.V.N., Emilia Cordero, R.N., Rabih Hijazi, M.D.* Farid Roman, M.D.* Paromita Datta*, Mariana Garcia Touza*; Indianapolis: Amale Lteif, M.D., Karen L. Moore, R.N., B.S.N, Christina Lazar-Robinson, M.D.*, Sanjay Gupta, M.D.* , M. Sue Kirkman, M.D.*, Martha Mendez, R.N.*, Zehra Haider, M.D.* , Lora Risley, R.N.*; Lexington: Dennis Karounos, M.D., Linda Barber, R.N., C.D.E., Janet Hibbard, B.S., James W. Anderson, M.D., L. Raymond Reynolds, M.D., Jeff Carlsen, M.D.*, Robert W. Collins, M.D.*, As’ad Ehtisham, M.D.*; Long Beach: Moti L. Kashyap, M.D., Barbara Matheus, RNP,MSN,CDE,BC-ADM, Tina Rahbarnia, B.S., Anthony N. Vo, M.D., Nancy Downey, M.S.N., N.P.*,.Lynette Fox, M.S.N., N.P.-C*, Richard M. Gonzales, M.D.*, C. Daniel Meyers, M.D.*, Subramaniam Tavintharan, M.D.*; Minneapolis: Frank Q.Nuttall, M.D., Ph.D., Lisa Cupersmith, R.N., Kathy Dardick, R.N.,.Linda Kollman, R.N., Angeliki Georgopoulos, M.D., Catherine Niewoehner, M.D.; Nashville: Stephen N. Davis, M.D., Paula Harper, R.N., C.D.E., Diana Davis, R.N., B.S.N., C.D.E., Jessica Devin, M.D., Annis Marney, M.D., Julia Passyn-Dunn, M.D., Jennifer Perkins, M.D., John Stafford, M.D.Al Powers, M.D.*,Linda Balch, R.N., C.D.E.* , Patricia Harris, R.N.* ,; Omaha : Robert J. Anderson, M.D.,.Diana Dunning, B.S.N., M.A., C.D.E. Steve Ludwig, R.N., Marlene Vogel, R.N., Cyrus DeSouza, M.D., Robert Ecklund, M.D.*, Sarah Doran, B.S.N.*, Claire Korolchuk, R.N.* , Mary McElmeel, B.S.N., M.S.*, Sarah Wagstaff, B.S.N.*; Phoenix: Peter Reaven, M.D., Bradley Solie, R.N., C.D.E., John Matchette, P.A.-C, Christian Meyer, M.D., Sylvia Vela, M.D., Nadeem Aslam, M.D.*, Eliot Brinton, M.D.*, Joy Clark, M.S.N, F.N.P-C, C.C.R.C., C.D.E.*,.Alisa Domb, R.N.*, Linda McDonald, R.N.*,.Lynae Shurtz, R.N., B.S.N.*; Pittsburgh: R. Harsha Rao, M.D., Janice N. Beattie, B.S.N., C.D.E., Carol Franko, C.R.N.P., Frederick R. DeRubertis, M.D.,.David Kelly, M.D*, Melisse Maser, C.R.N.P.*, Juleen Paul, CRNP, C.D.E.*; Richmond: Franklin Zieve, M.D., Ph.D., Susan J. Clark, R.N., M.S., C.C.R.C., Ann Grimsdale, R.N., Sonja Fredrickson, M.D., James Levy, M.D., Diane Schroeder, M.D.*; Salem: Ali Iranmanesh, M.D., Barbara Dunn, P.A.-C, Donna Arsura, R.N.,.Csaba Kovesdy, M.D., Suzanne Hanna, M.D.*Ashraf Iranmanesh, Pharm.D.*, Christy Florow* , Fe Remandaban, R.N.*, Erica Smith, L.P.N.*; San Diego: Robert R. Henry, M.D., Miriam Keller, R.N., B.S., Vanita Aroda, M.D., Charles Choe, M.D.,Steven Edelman, M.D.,

Andrea Gasper, PA-C,Dereck MaFong, M.D., Sunder Mudaliar, M.D., Deborah Oh, M.D., Rahil Bandukwala, M.D.*, Anna Chang, M.D.*, Sandeep Chaudhary, M.D.*, Sithophol Chinnapongse, M.D.*, Louie Christiansen, M.D.*, Neelima Chu, M.D.*, Dennis Kim, M.D.*,Mark Lupo, M.D.*, Chandran, Manju, M.D.*, Ray Plodkowski, M.D.*, Roopa Sathyaprakash, M.D.*, Janet Wilson, M.D.* ,Joseph Yu, M.D.*,Gina Macaraeg, R.N.*, Shelley Townes, R.N.*; San Antonio: Ralph DeFronzo, M.D.,.Lisa Johnson, M.S., RD/LD, Ken Cusi, M.D., Devjit Tripathy, M.D. Mandeep Bajaj, M.D.*,Janet Blodgett, M.D.*,.Sangeeta Kayshup, M.D.*, Mary Helen Vasquez, R.N., C.D.E.* , Barbara Walz, R.N., B.S.N., C.D.E.*, Tess Weaver, M.S.N., APRN., B.C., F.N.P.* ; San Juan: Julio Benabe, M.D., Zuleika Mercado, M.P.H., Brunilda Padilla, B.S.N., Jocelyn Serrano-Rodriguez, R.N., Carlos Rosado, M.D., Edwin Mejias, M.D.*, Tania Tejera, M.D.*, Clorinda Geldrez* , Elda Gonzalez-Melendez, B.S.N.*, Maria Natal, R.N.* Maribel Rios Jimenez, R.N.* ; Tucson: Jayendra H. Shah, M.D., Wendy S. Wendel, R.N., B.S.N., Lynnette Scott, R.N., Lynne A. Gurnsey, R.N. Fabia A. Kwiecinski, M.D., Thomas Boyden, M.D.*, Merilyn G. Goldschmidt, M.D.*, Virginia Easton*.

* Participants who left the VADT before its conclusion.
