# Supplementary material for: Predictors of Cognitive Decline in Older Adult Type 2 Diabetes from the Veterans Affairs Diabetes Trial
Source: Front Endocrinol (Lausanne). 2016 Sep 8;7:123. doi: 10.3389/fendo.2016.00123 (PMC5015004; doi:10.3389/fendo.2016.00123)
Supplement: Supplementary file 2 [file table_1.pdf]

Supplemental Table 1. Summary of baseline risk factors associated with 5-year decline in Trails- Making Part B test performance

Risk factor grouping	Parameter estimate	SE	N	P-value
Glucose-lowering medications				
Metformin (yes/no)	-0.457	0.171	1114	0.008
Medical co-morbidities				
Prior CV event (no/yes)	0.377	0.157	1084	0.016
Atherosclerosis				
Systolic bp (mm Hg)	-0.014	0.006	1106	0.011
Diastolic bp (mm Hg)	0.034	0.009	1106	< 0.001
Thrombosis				
Plasma fibrinogen	-0.002	0.0009	951	0.033
Aspirin use (yes/no)	-0.474	0.183	951	0.01
Diabetes-specific				
Diabetes duration (yrs)	-0.030	0.010	1075	0.004
Lipid-lowering medications				
Statins (yes/no)	-0.276	0.151	1114	0.068
Fibrates (yes/no)	0.126	0.213	1114	0.554

CV-cardiovascular; Bp- blood pressure
