# Supplementary material for: Predictors of Cognitive Decline in Older Adult Type 2 Diabetes from the Veterans Affairs Diabetes Trial
Source: Front Endocrinol (Lausanne). 2016 Sep 8;7:123. doi: 10.3389/fendo.2016.00123 (PMC5015004; doi:10.3389/fendo.2016.00123)
Supplement: Supplementary file 3 [file table_2.pdf]

Supplemental Table 2. Summary of baseline risk factors associated with 5-year decline in digit symbol test performance

Risk factor grouping	Parameter estimate	SE	N	P-value
Glucose-lowering medications				
Sulfonylureas (yes/no)	0.498	0.217	1155	0.022
Lipid-lowering medications				
Statins (yes/no)	0.083	0.177	1155	0.641
Fibrates (yes/no)	0.449	0.252	1155	0.075
Atherosclerosis/thrombosis				
Aspirin (yes/no)	-0.477	0.209	979	0.022
Diabetes- specific				
Diabetes duration (yrs)	-0.030	0.012	1112	0.015

SE- standard error
